# The Metabolomic-Gut-Clinical Axis of Mankai Plant-Derived Dietary Polyphenols

**DOI:** 10.3390/nu13061866

**Published:** 2021-05-30

**Authors:** Anat Yaskolka Meir, Kieran Tuohy, Martin von Bergen, Rosa Krajmalnik-Brown, Uwe Heinig, Hila Zelicha, Gal Tsaban, Ehud Rinott, Alon Kaplan, Asaph Aharoni, Lydia Zeibich, Debbie Chang, Blake Dirks, Camilla Diotallevi, Panagiotis Arapitsas, Urska Vrhovsek, Uta Ceglarek, Sven-Bastiaan Haange, Ulrike Rolle-Kampczyk, Beatrice Engelmann, Miri Lapidot, Monica Colt, Qi Sun, Iris Shai

**Affiliations:** 1 Faculty of Health Sciences, Ben-Gurion University of the Negev, Beer-Sheva 8410501, Israel; anatyas@post.bgu.ac.il (A.Y.M.); hila.zelicha@gmail.com (H.Z.); gtsaban@gmail.com (G.T.); ehudrinott@gmail.com (E.R.); alonkaplan47@gmail.com (A.K.); 2 Department of Food Quality and Nutrition, Fondazione Edmund Mach, Research and Innovation Centre, Via E. Mach, 1, San Michele all’Adige, 38098 Trento, Italy; kieran.tuohy@fmach.it (K.T.); camilla.diotallevi@fmach.it (C.D.); panagiotis.arapitsas@fmach.it (P.A.); urska.vrhovsek@fmach.it (U.V.); 3 Department of Molecular Systems Biology, Helmholtz Centre for Environmental Research GmbH, 04318 Leipzig, Germany; martin.vonbergen@ufz.de (M.v.B.); sven.haange@ufz.de (S.-B.H.); ulrike.rolle-kampczyk@ufz.de (U.R.-K.); beatrice.engelmann@ufz.de (B.E.); 4 Biodesign Center for Health through Microbiomes, School of Sustainable Engineering and the Built Environment, Arizona State University, Tempe, AZ 85281, USA; Dr.Rosy@asu.edu; 5 Department of Plant and Environmental Sciences, Weizmann Institute of Science, Rehovot 7610001, Israel; Uwe.Heinig@weizmann.ac.il (U.H.); asaph.aharoni@weizmann.ac.il (A.A.); 6 Biodesign Swette Center for Environmental Biotechnology, Arizona State University, Tempe, AZ 85287, USA; lzeibich@asu.edu (L.Z.); dcchang@asu.edu (D.C.); bedirks@asu.edu (B.D.); 7 Faculty of Science and Technology, Universitätsplatz 5-Piazza Università, 39100 Bozen-Bolzano, Italy; 8 Institute for Laboratory Medicine, University of Leipzig Medical Center, 04103 Leipzig, Germany; Uta.Ceglarek@medizin.uni-leipzig.de; 9 Research and Development Department, Hinoman Ltd., Rishon Lezion 7546302, Israel; miri@hinoman.com (M.L.); monica@hinoman.com (M.C.); 10 Department of Nutrition, Harvard School of Public Health, Boston, MA 02115, USA; qisun@hsph.harvard.edu; 11 Department of Epidemiology, Harvard School of Public Health, Boston, MA 02115, USA; 12 Channing Division of Network Medicine, Department of Medicine, Brigham and Women’s Hospital and Harvard Medical School, Boston, MA 02118, USA

**Keywords:** *Wolffia globosa*, polyphenols, flavonoids, plant-based nutrition, weight loss, mediterranean diet

## Abstract

Background: Polyphenols are secondary metabolites produced by plants to defend themselves from environmental stressors. We explored the effect of *Wolffia globosa* ‘Mankai’, a novel cultivated strain of a polyphenol-rich aquatic plant, on the metabolomic-gut clinical axis in vitro, in-vivo and in a clinical trial. Methods: We used mass-spectrometry-based metabolomics methods from three laboratories to detect Mankai phenolic metabolites and examined predicted functional pathways in a Mankai artificial-gut bioreactor. Plasma and urine polyphenols were assessed among the 294 DIRECT-PLUS 18-month trial participants, comparing the effect of a polyphenol-rich green-Mediterranean diet (+1240 mg/polyphenols/day, provided by Mankai, green tea and walnuts) to a walnuts-enriched (+440 mg/polyphenols/day) Mediterranean diet and a healthy controlled diet. Results: Approximately 200 different phenolic compounds were specifically detected in the Mankai plant. The Mankai-supplemented bioreactor artificial gut displayed a significantly higher relative-abundance of 16S-rRNA bacterial gene sequences encoding for enzymes involved in phenolic compound degradation. In humans, several Mankai-related plasma and urine polyphenols were differentially elevated in the green Mediterranean group compared with the other groups (*p* < 0.05) after six and 18 months of intervention (e.g., urine hydroxy-phenyl-acetic-acid and urolithin-A; plasma Naringenin and 2,5-diOH-benzoic-acid). Specific polyphenols, such as urolithin-A and 4-ethylphenol, were directly involved with clinical weight-related changes. Conclusions: The Mankai new plant is rich in various unique potent polyphenols, potentially affecting the metabolomic-gut-clinical axis.

## 1. Introduction

Plants produce secondary metabolites known as polyphenols in order to defend themselves from stressors such as insect herbivores, microbial infection and UV light [1]. Polyphenol bioavailability is affected by environmental factors, storage conditions and cooking methods [2]. Across ~8000 polyphenolic structures described, the main groups are classified by the number of phenol rings they contain and structural elements. They include phenolic acids, flavonoids, stilbenes and lignans [3]. Among the 100 richest dietary sources of polyphenols are cloves, cocoa powder, black olives, green tea, extra virgin olive oil, different berries, nuts and wine [4]. Due to their antioxidant property, dietary polyphenols may have a beneficial effect on human and animal health, including liver protection, anticancer activities [5], reduced cardiovascular risk [6] and reduced inflammation [7]. The Mediterranean (MED) diet’s polyphenol content is relatively high due to its wide range of plant food sources [8]. In the traditional Spanish MED diet, the mean polyphenol intake was estimated to be between ~2500–3000 mg/day [9] compared with ~1000 mg/day in a western-style diet [10].

A small portion (5–10%) of polyphenols and their metabolites are absorbed in the upper gastrointestinal tract, depending on their structure [11]. In the small intestine, some dietary polyphenols that cannot be absorbed in their native form undergo enzymatic modifications (e.g., methylation, esterification). In contrast, the vast majority of dietary polyphenols reach the large intestine, where they undergo microbial metabolism [2,12]. The metabolites resulting from these processes can then be up taken via the portal vein into the liver, and then via the circulation to other organs and tissues for further utilization before being excreted in urine [13]. In both plasma and urine, the detection of polyphenols or their derivatives depends on the amount of ingestion, their chemical structure and the extent of their microbial biotransformation. In both biofluids, metabolite concentrations change dramatically over time after ingestion, with polyphenols of high bioavailability peaking in blood in the first hours after ingestion before being cleared from the blood into the urine during excretion [14]. On the contrary, poorly bioavailable polyphenols, which undergo extensive microbial biotransformation, peak in blood about 7 to 10 h after ingestion before being cleared from the blood and excreted in urine [15].

*Wolffia globosa* ‘Mankai’ is an aquatic plant of the duckweed family recently identified for its nutritional value [16,17]. It is a source of omega-3 fatty acids [18], dietary fiber, polyphenols [19] and, as reported by us, a rich source of protein [20], iron [21], vitamin B12 [22] and folate. Consuming one cup of Mankai shake exclusively provided in the DIRECT PLUS trial, equivalent to ~20 g of dry matter, provides the following proportions of recommended intakes: 18% whole bioavailable protein, 75% bioavailable iron, 60% folic acid and 21% vitamin B12. In this study, we explored the polyphenol content and manifestation of the Mankai aquatic plant, the smallest plant on earth (~0.5 mm), with the highest surface area to volume ratio, and exposed to both air and aquatic mediums. We also evaluated whether polyphenols in the diet, including polyphenols from Mankai, could be detected in human plasma and urine.

## 2. Materials and Methods

### 2.1. Plant Metabolites and Polyphenol Detection and Identification

We used several laboratory methods to detect plant polyphenol metabolomics, performed in three different centers (further methodology details for all experiments are presented in Methods S1). Briefly, analyses are presented as Mankai polyphenols metabolomic experiments 1–5, according to the following methods and procedures.

Mankai polyphenol metabolomics Experiment 1 performed at Helmholtz Center for Environmental Research (UFZ), Germany. All the chemical screening was carried out with a High-Performance Liquid Chromatography Quadrupole Time of Flight (HPLC-QToF) instrument from Agilent Technologies (6540 UHD Accurate-Mass Q-ToF Liquid chromatography-mass spectrometry (LC/MS) instrument; Santa Clara, CA, USA). Every sample was injected twice to be ionized in positive and negative modes. 10 µL of the sample was injected and loaded on a C18 column (an ACQUITY UPLC HSS T3, 2.1 × 100 mm column). The compounds were separated with a gradient of mobile phase A (0.1% formic acid in water) and mobile phase B (2% isopropanol, 0.1% formic acid in acetonitrile). The gradient was as follows: 0–5 min 1% B, 5.1–20 min 1%-100% B, 20.1–22 min 100% B, 22.1–25 min 1% B. The QToF was set up in centroid mode and in screening mode, allowing the detection of ions with a mass to charge ratio between 60 and 1000. Ions with intensities above 200 counts was sent to the second MS to record their fragments.

Mankai polyphenol metabolomics Experiment 2 performed at UFZ, Germany. LC-Orbitrap MS HPLC-column and gradient were performed as stated above, but on an Acquity Ultra Performance LC (Waters, Waters Corporation, Milford, CT, USA). The HPLC was coupled to an Orbitrap Velos mass spectrometer (Thermo Fisher Scientific, Waltham, MA, USA) with a heated electrospray source. ESI heater temperature was set to 300 °C. A full scan was acquired for *m*/*z* between 60 and 1000. The ten most intense signals were fragmented using CID with a collision energy of 35. Samples were measured in negative and positive ionization mode separately. For experiments, 1 and 2, extraction of the samples was performed following the protocol from De Vos et al. [23].

Mankai polyphenol metabolomics Experiment 3 performed at the Weizmann Institute, Israel using LC/MS. Plant material was separated from the medium by filtration through two layers of Miracloth. After washing with ice-cold water and gentle drying, the material was shock-frozen and stored at −80 °C until analysis. Tissue was then homogenized frozen with a TissueLyser Retsch mill (Retsch, Hahn, Germany; 20 Hz for 2 min, twice) and extracted with 80% MeOH + 0.1% formic acid (weight per volume 1:3) as described earlier ([24] and Methods S1). Analysis was performed using an Ultra Performance Liquid Chromatography (UPLC)-QToF system (HDMS Synapt, Waters) with the following chromatographic conditions: column: 100 × 2.1-mm i.d., 1.7-μm UPLC BEH C18 column (Waters), phase A: 5%ACN + 0.1% formic acid, phase B: 100% ACN + 0.1% formic acid, t0: 0 min 0% B, 0–22 min 28% B, 22–36 min 100% B, 36–38 min 100% B, 38–38.5 min 0% B, 38.5–40 min 0% B. The mass spectrometer was operated in centroid MS^E^ mode, acquiring, in parallel, mass spectra with CE 4 eV, and a second channel mass fragmentation spectra using a collision energy ramp, 10–30 eV in positive ionization mode and 15–35 eV in negative ionization mode.

Mankai polyphenol metabolomics Experiment 4 performed at Edmund Mach Foundation, Italy. Three equal samples of Makai in the form of powder were analyzed. The three samples did not show any major differences in their metabolic fingerprint. For extraction, 0.1 g of Makai powder was extracted with 1 mL MeOH/H_2_O/formic acid (75/24.9/0.1) for 1 h at room temperature with an orbital shaker, centrifuged for 5 min and finally filtered and directly analyzed. A Synapt G1 UHPLC-DAD-QToF-MS (Waters) was used for the analysis of the samples and the annotation of the major peaks, according to previously published methods [25,26] (https://doi.org/10.1007/s11306-014-0638-x and https://doi.org/10.1002/rcm.6705 accessed on 10 May 2021).

Mankai polyphenol metabolomics experiment 5. LC-MS/MS performed at Edmund Mach Foundation, Italy using 10 different Mankai samples (differed by season and exposure to light). Phenolic compounds in the Mankai plant and lyophilized sample were determined according to a previously published method [27], with some modifications. Details of the liquid chromatography and mass spectrometry have been described before [27,28].

### 2.2. Anoxic Gut Bioreactors Mankai Experiment Performed at Biodesign Swette Center for Environmental Biotechnology, Arizona State University, USA

Microbiota Reactors (Human Fecal Mixture) & Media, Anoxic Bioreactor, Mankai Lysate, and Sampling: The procedure of preparing the reactors (artificial gut) is detailed elsewhere, along with the chemical and molecular analyses [22].

### 2.3. Polyphenols in Human Plasma and Urine. The 18-Month DIRECT-PLUS Clinical Trial, Performed at Ben Gurion University, Israel

#### 2.3.1. Study Design

The 18-month long DIRECT-PLUS trial (clinicaltrials.gov ID: NCT03020186) was performed in an isolated workplace in Israel. This site includes a medical department and cafeteria with monitored lunch. In this trial we aimed to address the residual beneficial effect of a green Mediterranean diet, richer in green plants and lower in meat, compared with other healthy lifestyle strategies. Inclusion criteria were age above 30 years with abdominal obesity or dyslipidemia. Exclusion criteria and ethical issues are described elsewhere [22,29,30].

#### 2.3.2. Randomization and Intervention

Randomization and intervention are described elsewhere [21]. Briefly, participants were randomly assigned to one of three intervention groups, all combined with physical activity recommendation and a free gym membership:

Healthy dietary guidelines (HDG) group. The participants received basic health-promoting guidelines for achieving a healthy diet.

Mediterranean (MED) group. Participants were instructed to adopt a calorie-restricted Mediterranean diet as described in our previous trials: DIRECT [31] and CENTRAL [32] trials, supplemented with 28 g/day of walnuts (containing 440 mg polyphenols/day; gallic acid equivalents (GAE) [33]).

Green Mediterranean (green-MED) group. The calorie-restricted green-MED diet was restricted in processed and red meat and was richer in plants and polyphenols. In addition to 28 g/day of walnuts, as in group MED, the participants were guided to consume the following items further: 3–4 cups/day of green tea and 100 g/day of Mankai (*Wolffia globosa* ‘Mankai’ cultivar) plant as frozen cubes, which together provided additional daily intake of 800 mg polyphenols (GAE). Details regarding the lifestyle interventions and motivation techniques are detailed elsewhere [30]. Mankai, green tea and walnuts were provided free of charge and monitored at the on-site clinic.

#### 2.3.3. Human Samples and Polyphenol Analysis

Blood and urine samples were taken at 8:00 AM after a 12-h fast, at baseline, and after six and 18 months of intervention. The blood samples were centrifuged and stored at −80 °C. The determination of plasma polyphenol metabolites is detailed elsewhere [30], and in Methods S2. Briefly, the analysis was performed according to Vrhovsek et al. [27,28], with some modifications, at the Department of Food Quality and Nutrition, Research and Innovation Centre, Fondazione Edmund Mach, Trento. Italy. Only ten polyphenols were detected in plasma, and data regarding these polyphenols are reported as detected/not detected. Urine polyphenols analysis was performed at UFZ (Methods S3). The 140 identified polyphenols are reported here as relative to baseline intensities (area under the curve). The samples from the same participants were handled identically and assayed in the same batch to avoid systematic biases. Details regarding other measurements, including anthropometric parameters, electronic questionnaires and blood biomarkers, are available elsewhere [29,30].

#### 2.3.4. Statistical Analysis

Continuous parameters are presented as means and standard deviation (SD). Plasma polyphenols are measured as “detected/not detected” numbers and/or percentages. Urine polyphenols are presented as log-transformed change and presented as relative intensities. Correlations were examined using the Spearman or Pearson test according to the variable’s distribution, determined by the Shapiro Wilk test. An Analysis of Variance (ANOVA) test was performed to compare the three intervention groups with a post hoc Tukey test. Correction for multiple comparisons (for more than three comparisons) was applied using the Benjamini-Hochberg correction with a False Detection Rate (FDR) of 5–25% (according to the phase of the analysis, used to avoid excessive filtering). Data from electronic questionnaires were reported as increased/decreased/same intake in the months prior to the administration [21]. The comparison between nominal parameters (e.g., detected/not detected polyphenol, change in food item intake) was performed using the Chi-square test. Differences were considered significant for *p* < 0.05. Statistical analyses were performed using R software, version 4.0.3.

## 3. Results

### 3.1. Mankai Plant Metabolites

Overall, 198 different phenolic metabolites were detected in all experiments, mainly from the flavonoids and phenolic acids classes. Examples of the polyphenols found in the Mankai plant are presented in Table 1.

In the first set of experiments (experiments 1 + 2), we identified in Mankai samples a total of 90 phenolic candidates according to the Orbitrap technique, and 57 phenolic candidates in the QTof analysis. Among phenols detected were scutellarin, isovitexin, ginkgolic acid, daphnetin, cinnamic acid, and bergenin (Orbitrap analysis); rutin, orientin, and apiin (QToF).

**Figure 1:**
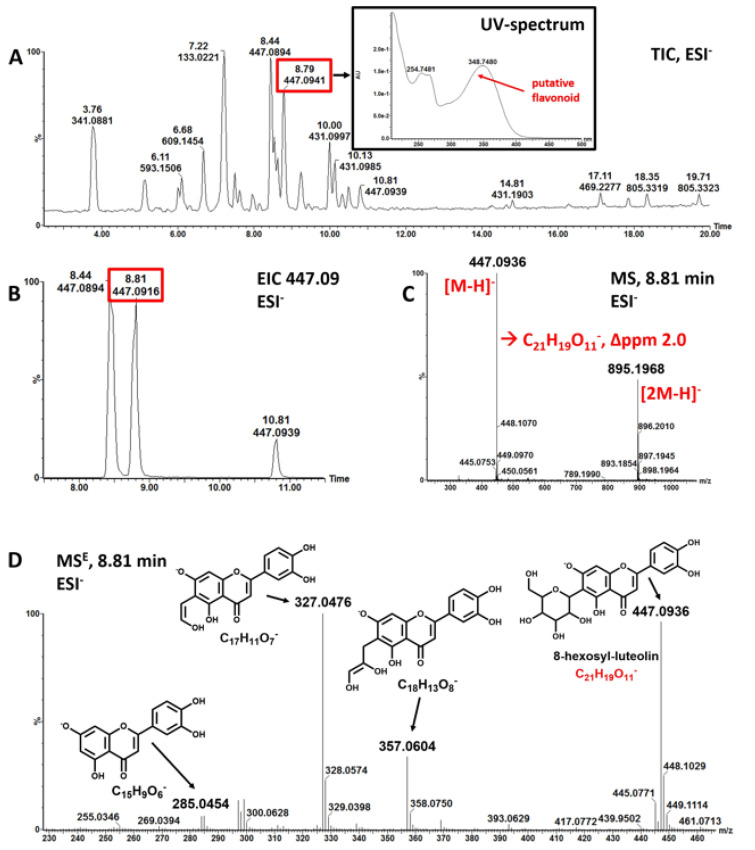
(**A**–**D**) Putative identification of polyphenolic compounds from LC-MS measurements (experiment 3). A. total ion chromatogram (TIC) of *Wolffia globosa* ‘Mankai’ extract acquired in negative ionization mode (ESI-); inlay: UV-absorption spectrum of compound eluting at 8.8 min. B. extracted ion chromatogram of mass-to-charge ratio (*m*/*z*) 447.09. C. background corrected mass spectrum of peak at retention time 8.81 min; two masses are detected *m*/*z* 447.0936 assigned as [M-H]^−^ and the dimer of 447.0936, m/z 895.1968 assigned as [2M-H]^−^; elemental composition of the ion m/z 447.0936 was calculated to C_21_H1_9_O_11_- with a mass error of 2 ppm. The molecular formula corresponds to putative 8-hexosyl-luteolin. D. mass fragmentation spectrum acquired in MS^E^ mode (MS^E^) spectrum of m/z 447.0936 confirms assignment as 8-C-hexosyl-luteolin. Major fragments are shown with structure and elemental composition. EIC: extracted ion chromatogram.

Next, we examined polyphenols in Mankai methanolic extracts of fresh plant material that were most similar to the plant material used for the dietary study were identified using a nontargeted workflow (experiment 3). We aimed to assign the major, most abundant polyphenols in the metabolic profile, assuming that these compounds are the major contributors to polyphenol related dietary effects. Using this workflow (Figure 1A–D and Figure S1), we putatively assigned the 29 most prominent polyphenolic compounds (comparison to compounds in the in-house natural product library [34] and to other assigned compounds in the Wolffia extract); mainly luteolin, apigenin, and quercetin derivatives (Table S1).

**Figure 2:**
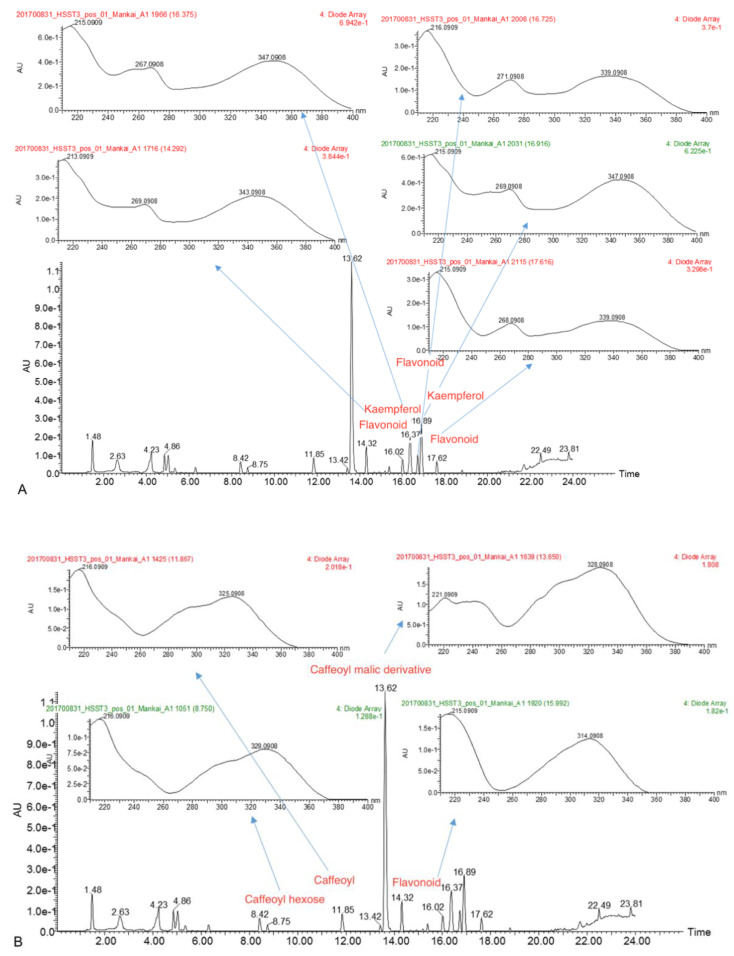
(**A**,**B**) Example of a UV spectra of the metabolites (experiment 4). (**A**). Flavonoid group quercetin and kaempferol derivatives. (**B**). Cinnamic group caffeoyl and coumaroyl derivatives.

In the next analysis (experiment 4), performed in three Mankai samples, we identified two metabolites (data compared with an in-house library) annotated to kaempferol and quercetin (Figure 2A). The following putatively characterized compound classes were also detected: flavonoids, caffeoyl hexose, caffeoyl’s derivatives, and coumaroyl hexose (Figure 2B). Further analysis in these samples using XEVO UHPLC-MS/MS showed 35 different polyphenols (including quercetin, rutin, luteolin, caffeic acid and daphnetin). This laboratory performed an additional experiment with ten other Mankai samples (experiment 5) using the LC-MS/MS technique, with similar polyphenols detected (a total of 26 polyphenols, including quercetin, catechin, caffeic acid, apigenin, and luteolin).

### 3.2. Gut Degradation Products

**Figure 3:**
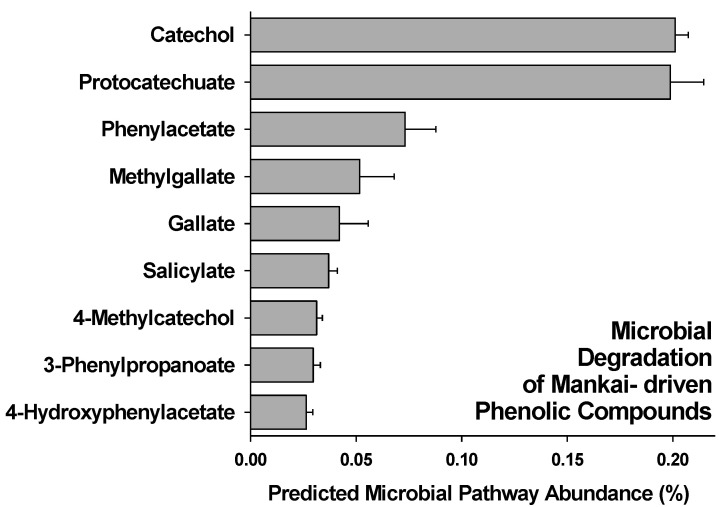
Relative abundance of predicted microbial pathways in Mankai-supplemented artificial gut bioreactors.

Predicted functional profile analysis via PICRUSt [35] at the end of incubation (day 14) showed that Mankai-supplemented artificial gut bioreactors displayed a significant higher relative abundance of 16S rRNA gene sequences belonging to bacteria that have genes in their genomes that encode for enzymes for nine microbial pathways involved in the degradation of Mankai-derived phenolic compounds (Figure 3). Especially, the pathways for degradation of (a) protocatechuate, a phenolic acid, and (b) catechol, also known as 2-Hydroxyphenol, were predictively more abundant in Mankai-supplemented bioreactors. In control bioreactors that lacked Mankai, none of these sequences associated with the microbial degradation of phenolic compounds were detected.

### 3.3. Polyphenols Detection in Human Plasma and Urine

#### 3.3.1. DIRECT PLUS Baseline Characteristics

The mean body mass index (BMI) of the participants was 31.3 ± 4.0 kg/m^2^, and the mean WC was 110.6 ± 9.1 cm for men and 103.3 ± 9.6 cm for women. All baseline characteristics (e.g., weight: 93.7 ± 14.3, WC: 109.7 ± 9.5, systolic blood pressure: 130.3 ± 14.0, diastolic blood pressure: 81.1 ± 10.2; data presented for the entire study population) did not differ between the intervention groups [29,30]. Ten polyphenols were detected in the participants’ baseline plasma samples, with no significant differences between groups (Table 2). We detected in more than 50% of the plasma samples the following polyphenols: hippuric acid, m-hydroxyhippuric acid, and vanillin.

#### 3.3.2. Plasma Polyphenols following 18 Months of DIRECT PLUS Weight Loss Intervention

After 18 months (89.8% subject retention rate) higher and similar weight loss was observed, following a caloric deficit, in the two MED groups (MED: −2.9 ± 5.2%; Green-MED: −3.9 ± 6.5%) compared with the HDG group (−0.6 ± 5.1%, *p* < 0.05 for both MEDs vs. HDG). As previously reported, at baseline there were no differences between the intervention groups in the intake of macronutrients and specific food items [30]. After six and 18 months, the green-MED diet group significantly increased the intake of Mankai and green tea (*p* < 0.001 for both provided items between intervention groups; mean 18-month weighted intake in the green MED group: 2.8 cups/day of green tea and 2.6 Mankai shakes/week [30]), compared to the other groups, and both MED groups significantly increased intake of the provided walnuts (*p* = 4.4 × 10^−8^) compared with the HDG group. The HDG showed the greatest increase in fruit intake compared to the other MED groups (*p =* 0.007). No difference in vegetable intake between the groups was reported (*p* = 0.08).

**Figure 4:**
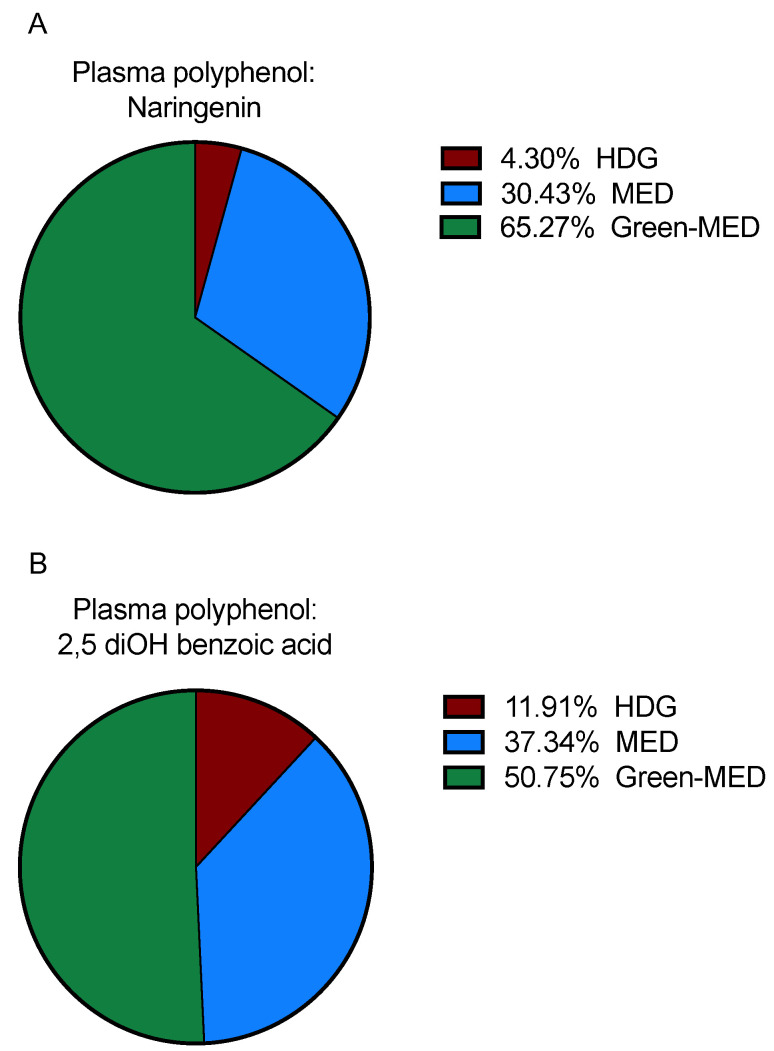
(**A**,**B**) Differentially detected plasma polyphenols between groups at the end of the intervention. A: Naringenin: out of the three groups, the highest detection was among the green-MED dieters (65.27% of all detection in whole DIRECT PLUS samples), followed by the MED (30.43% detection) and HDG (4.3% detection) groups; *p* = 0.001. B: 2,5 diOH benzoic acid: green-MED group showed that highest detection (detection of 50.75%), followed by the MED (37.34%) and HDG (11.91%); *p* = 3.7 × 10^−5^. Differences between groups were calculated using the Chi-square test. HDG, Healthy dietary guidelines; MED, Mediterranean.

According to the follow up data, four polyphenols were differentially detected in plasma between the groups at the end of the intervention, mainly naringenin and 2,5 diOH benzoic acid (Figure 4A,B). In contrast, other plasma polyphenols were similarly detected among all groups: vanillic acid (*p* = 0.65), hippuric acid (*p* = 0.88), m-hydroxy hippuric acid (*p* = 0.51), diOH isoferulic acid (*p* = 0.27), v (*p* = 0.99), and pyrogallol (*p* = 0.13).

#### 3.3.3. The Effect of the Intervention on Urine Polyphenols

**Figure 5:**
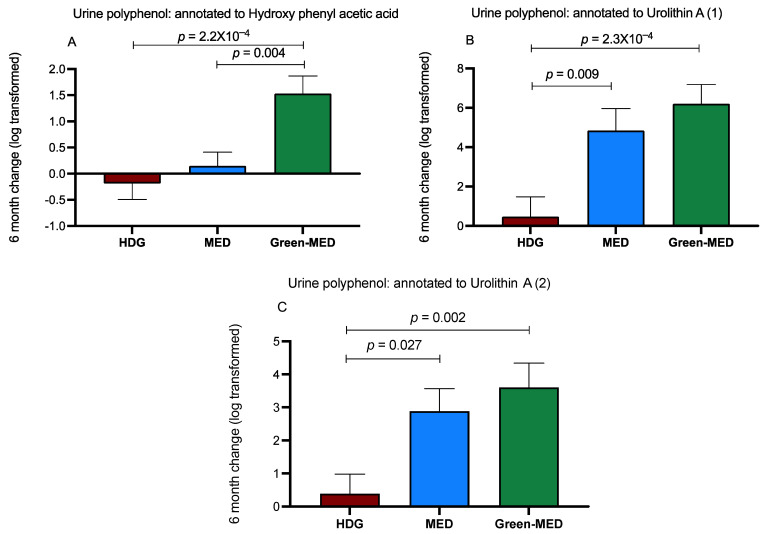
(**A**–**C**) Differential six-month change (relative change, log-transformed) of urine polyphenols, between-group comparisons. Between-group changes are corrected for multiple comparisons. Data presented as means of log change and SEM. A: Urine polyphenol annotated to 6-month hydroxy phenyl acetic acid: *p* = 1.5 × 10^−4^, *q* = 1.7 × 10^−3^. B: Urine polyphenol annotated to 6-month urolithin A: *p* = 2.9 × 10^−4^, *q* = 3.6 × 10^−3^. C: Urine polyphenol also annotated to 6-month urolithin A: *p* = 0.002, *q* = 0.007.

As opposed to the plasma, when examining urine samples of the participants, 140 phenolic compounds annotated to 75 polyphenols were detected (Table S2). We observed that after six months, the relative intensity changes in 16 polyphenols significantly differed between the intervention groups. Further correction for multiple comparisons resulted in few polyphenols that showed a significant incremental difference between groups after six months (Figure 5A–C). The 18-month change in urine hydroxy phenyl acetic acid also showed an incremental increase upward across groups (*p* = 0.023 between HDG and green-MED).

#### 3.3.4. Polyphenols and Health Outcomes

We examined correlation in the acute phase of the intervention (first six months) between 6-month changes in anthropometric (weight, WC, systolic and diastolic blood pressures, glycemic (fasting glucose and insulin) and lipid (total cholesterol, triglycerides, HDLc, and low-density lipoprotein cholesterol (LDLc) markers with acute changes in urine polyphenols. After correcting for multiple comparisons (total of 1400 comparisons, 25% FDR), we observed several significant correlations, with the strongest correlation observed between WC change and 4-ethylphenol change (*r* = −0.261, *p* = 1.2 × 10^−5^, *q* = 1.8 × 10^−4^). Urolithin A was the most prominent polyphenol with significant correlations with weight (*r* = −0.259, *p* = 1.3 × 10^−5^, *q* = 3.6 × 10^−4^) and WC (*r* = −0.258, *p* = 1.5 × 10^−5^, *q* = 5.4 × 10^−4^) changes. These correlations of 4-ethylphenol and urolithin A with weight and WC remained significant after stricter filtering (5% FDR). Changes after 18 months in these two polyphenols showed the same pattern (*r* = −0.158, *p* = 0.011 for the correlation between urolithin A and weight; *r* = −0.164, *p* = 0.009 for the correlation between 4-ethylphenol and WC).

Finally, we examined the correlation between the polyphenols that showed a significant increase in the Green-MED group with serum folic acid levels (an objective marker of green leaf intake). We found a significant correlation of the following polyphenols (6-month relative change, three intensities change annotated to two polyphenols) with the 6-month change in serum folate: hydroxy phenyl acetic acid (*r* = 0.136, *p* = 0.024) and urolithin A (two annotations: *r* = 0.158, *p* = 0.009; *r* = 0.193, *p* = 0.001).

## 4. Discussion

The present study found that the Mankai plant contains approximately 200 polyphenols and phenolic compounds, most of the flavonoid class. We also discovered that Mankai incubation in an anoxic gut resulted in a higher relative abundance of microbial phenolic compound degradation pathways compared with control. Among participants undergoing diets differing by their polyphenol content, we detected polyphenols in plasma and phenolic degradation products in urine samples following months of intervention. The participants who consumed Mankai as part of their assigned diet, and other polyphenol-rich products such as green tea and walnuts, had higher plasma and urine polyphenol levels than other groups. The increase in urine polyphenols was correlated with beneficial effects, mostly with better anthropometric parameters. Finally, Mankai intake, represented by serum folic acid level, was associated with an increase in several urine polyphenols. Overall, our findings suggest that the Mankai new plant is rich in various unique potent polyphenols, potentially affecting the metabolomic-gut clinical axis.

Several limitations should be considered. First, plasma polyphenol analysis might be limited due to biological and metabolism-related factors, such as the amount of polyphenols ingested, the specificity of the metabolite, the nature of the polyphenol, and the fact that fasted plasma samples (at least 12 h after the last Mankai ingestion) were used for the analysis, a time when most polyphenols derived metabolites would have already been cleared from the blood [36]. Thus, plasma polyphenol data are presented as a dichotomous parameter (detected/not detected) rather than a continuous one. We completed the plasma polyphenols results with urine polyphenols analysis. These are also limited since this analysis was qualitative in the form of intensities rather than concentrations, presented as relative to baseline log-transformed change. In addition, the urine polyphenol analysis was based on a spot sample rather than a 24-h collection, although these samples may have contained metabolites of polyphenols taken at the dinner. Second, our bioreactor data are from a lab-based condition; thus, an open question remains concerning whether the Mankai plant may modify the microbiota in the intestinal tract with a possible effect on polyphenol degradation products. Moreover, the generalizability of the findings is in question, since the human gut microbiota consists of many species that are not culturable, so whether the same findings can be observed in free-living humans is yet to be explored. Finally, we could not isolate the green-MED diet’s specific components, as this was administered for an extended period. The laboratory tests were held following the whole diet’s consumption and not following a specific component’s test meal. Since we provided several types of polyphenol-rich foods, such as green tea and walnuts, we cannot determine which phenolic metabolites in plasma or urine originated from Mankai, and which originated from other foods. However, objective measurements such as serum folic acid allowed us to correlate them with urine polyphenols. The strengths of the current series includes combining results on plant metabolites from several unbiased laboratories and different Mankai batches, with the results complementing each other, gut-related analysis, and data from a long-term, large scale, human randomized controlled trial with monitored lunch and free daily supply of Mankai and other polyphenol-rich foods to the participants throughout the trial period. Here, we tried to explore the new plant polyphenols manifestations in a wide comprehensive range of methods.

In the experiments investigating the phenol metabolomics of the Mankai plant, we observed around 200 polyphenols and phenolic metabolites. Previous studies suggested that the *Wolffia globosa* plant has high phenolic and antioxidant content [19], with a high concentration of the flavonoid class polyphenols Luteolin and Apigenin derivatives [37]. Quercetin, apigenin, rutin, and luteolin (all flavonoids) were detected in most of our laboratory experiments, as well as daphnetin (classified as other polyphenols) and caffeic acid (phenolic acid). The large group of flavonoids is divided into subclasses that include, but are not limited to, flavanols (e.g., catechins), flavones (e.g., rutin and luteolin), flavonols (e.g., quercetin), and flavanones (e.g., naringenin), and are integral components in our diet [5]. Previous studies recognized the beneficial effect of flavonoids on health due to biological activities that include antioxidation, liver protection and anti-inflammation, and some studies suggest anticancer activity [5]. Phenolic acids, specifically hydroxycinnamic acids as caffeic acid, were demonstrated, mostly in animal studies, as antiatherosclerosis, antidiabetic, and effective against Alzheimer’s disease, other brain dysfunctions, liver and kidney injuries and skin cancer [38]. Coumarins, such as daphnetin, generally used as additives in food and in the cosmetic and pharmaceutical industries, have been associated with reducing the risk of cancer, diabetes, cardiovascular and brain diseases [39]. These experiments further contribute to the currently limited knowledge regarding *Wolffia globosa* phenolic compound content, and specifically to the *Wolffia globosa* ‘Mankai’ cultivar’s unique phenolic profile.

The relationship between polyphenols and the gut microbiota is suggested to be two-way. Intestinal bacteria have a fundamental role in the digestion of polyphenols, and the bioactivity and bioavailability of dietary polyphenols might be affected by some gut bacteria [40]. However, this might also be the other way around, with dietary polyphenols altering the gut microbiota community. Based on genome prediction from 16S rRNA gene sequences of microorganisms in anoxic bioreactors inoculated with human fecal samples, predicted functional pathway analysis indicated that at least some Mankai-derived phenolic compounds are metabolized by the human gut microbiota, and thus degraded before human absorption. Although is it widely accepted that plant-derived phenolic compounds, such as protocatechuate, phenylacetate, methylgallate and salicylate have anti-inflammatory, antioxidant, and antitumor properties, and thus have the potential to improve human diseases such as coronary heart diseases, cancer and diabetes [41,42,43,44,45], some plant-derived phenolic compounds, such as catechol and methylcatechol, can have cytotoxic and cancerogenic activities in the human body [46,47]. However, intracerebroventricular 4-methylcatechol has the potential to increase spatial learning and memory and produce an antidepressant effect [48]. The pathways for degradation of catechol and protocatechuate were predictively more abundant in Mankai-supplemented bioreactors. Catechol is a biodegradation product of phenolic compounds in microorganisms [49], and protocatechuate, also known as protocatechuic acid, is one of the most abundant catabolites in the large intestine, and derives from different polyphenols (e.g., quercetin glycosides) [50]. Catechol and protocatechuate are central intermediates in the aerobic peripheral pathways of the degradation of aromatic compounds originated from, for example, cinnamate and caffeate [51]. Thus, the bioreactor results suggest that gut microbes utilize at least some of the Mankai-derived phenolic compounds. Based on the assumption that some of these compounds are harmful to health, microbial degradation—before human absorption—has a potential detoxifying effect. However, these preliminary results do not allow any quantitative conclusions about the microbial usage of plant-derived phenolic compounds, which warrants further studies.

The DIRECT PLUS intervention trial aimed to examine how incremental amounts of dietary polyphenols affect different health outcomes. On top of the Mediterranean diet, based on vegetables, fruits and nuts, all naturally containing high amounts of polyphenols, we provided 28 g a day of walnuts in both MED groups, as well as 3–4 cups of green tea and one Mankai shake in the green-MED group. In this study, we demonstrated the polyphenols found in Mankai. Our previous work described the beneficial effect of Mankai on glycemic response [52], cardiovascular risk [29], the gut microbiome [53] and liver steatosis [30]. Our previous and current observations regarding the Mankai plant may contribute to better understanding the effect of this plant on health. The main polyphenols in walnuts are ellagitannins, ellagic acid, and its derivatives [33]. Walnuts are considered to have a beneficial effect on health maintenance and disease prevention [54]. Ellagitannin found in nuts was reported to reduce WC, LDLc, and triglycerides [55]. Most of the polyphenols found in green tea are catechins, mainly epigallocatechin (EGC), epicatechin gallate (ECG), and epigallocatechin gallate (EGCG) [2,56]. Health benefits of green tea as a drink, or in the form of extract, include improvement in cardiometabolic health [57,58], weight reduction [56] and improved cognitive function [59,60]. Results from this study show that while at baseline the detection of 10 plasma polyphenols was similar across the intervention groups, in polyphenols that significantly differed between groups at the end of the intervention, the MED groups showed higher detection compared with the group actively receiving the least polyphenols. The flavanone naringenin demonstrated a beneficial effect on cardiometabolic risk reduction in humans (e.g., reduction in blood glucose and increase in HDLc) [61], which showed the highest detection in the green-MED group. 2–5 dihydroxybenzoic acid, a phenolic acid known to be a catabolite of the plant hormone salicylic acid [62], was also the highest in the plasma green-MED dieters compared with the other groups. Although we detected small amounts of polyphenols in the participants’ plasma, these results may further establish an unbiased assessment method for the intake of dietary polyphenols.

Urine polyphenol analysis showed a similar pattern to plasma analysis, with some incremental detection between the intervention group: 6-month hydroxy phenyl acetic acid and urolithin A. An 18-month change in hydroxy phenyl acetic acid showed this trend as well. Urolithin A is one of the degradation products of ellagic acid, found in both Mankai and walnuts, resulting from gut microbiota activity [63]. An elevation in urine urolithin A among individuals with metabolic syndrome was observed following 30 g/day of walnuts intake for 12 weeks and was suggested as a marker for walnuts intake [64]. Urine urolithin A was correlated with blood cardiometabolic biomarkers in overweight and obese adults, and thus suspected to be cardioprotective [65]. In this study, urolithin A, apart from showing an incremental trend upwards in the most polyphenol-rich diet, was also inversely correlated with weight and WC changes. Similarly, 4-ethylphenol, a potential urine biomarker for quercetin intake [66], was also inversely correlated with WC changes. Since weight changes might reflect adherence to the intervention, and thus associated with polyphenol change, these correlations should be interpreted with caution. Hydroxyphenyl acetic acid also showed a trend upwards with increasing polyphenol intake, according to the intervention groups, and is the urine end product of catechins [67]. Another study suggested that hydroxyphenyl acetic acid is a fecal deconjugated quercetin derivative [68]. Urine hydroxyphenyl acetic acid metabotypes such as 3,4 hydroxyphenyl acetic acid (a urine metabolite of ferulic acid [69], quercetin, and rutin [70]) were elevated following green tea consumption [67]. It has to be noted that the amount of the ingested food containing polyphenols might lead to an unclaimed interaction, synergism, and/or inhibition between bioactive compounds such as peptides or other antioxidants (e.g., ascorbic acid) [71,72]. This could affect digestion, absorption, metabolism and (biological) function of molecules. Similarly, as with other whole plant foods rich in complex polyphenols, the underlying mechanisms of action might include nutrient:nutrient and nutrient:host enzyme interactions in the gut, as well as the more recognized biological activities of polyphenols and their derivatives upon absorption. The direct effect of complex polyphenols on digestive enzymes, nutrient absorption, intestinal redox potential and intestinal inflammation are all important and likely contributors to the metabolic and immune related health effects of plant polyphenols [73]. Furthermore, the molecular weight of the polyphenol, the number of hydroxyl or galloyl groups, and their position, might be related to the antioxidative activity of the polyphenol. For example, an additional hydroxyl group at the para-position might increase antioxidant activity [72]. In this study, we could not determine these effects. Our human intervention, DIRECT-PLUS, was designed to demonstrate cause and effect between ingestion of a Green Mediterranean diet supplemented with Mankai and metabolic health and provided insight into physiological effects related to body fat composition/partitioning, cognitive function and blood biochemistry. Future mechanistic studies examining direct effects of Mankai and other whole plant foods on the gut and, indeed, the gut microbiota, are needed to measure the relative contribution of gut-specific, as opposed to system effects of dietary polyphenols. Overall, the observed increase in urine phenolic metabolites provides additional evidence that dietary polyphenols might be traceable in urine and indicate polyphenol-rich foods.

## 5. Conclusions

This study provides a complementary view of polyphenol-rich foods’ metabolome-gut-clinical axis, taking Mankai as a case study. Considering the latest evidence regarding the nutritional profile of the aquatic plant Mankai [20,22], the acceptance among our participants in the DIRECT PLUS trial, and current knowledge regarding its effects as part of green-MED diet on fatty liver, glycemic control, cardiometabolic risk and the gut microbiome [29,30,52,53], it can potentially offer a green, healthy meat substitute. However, further studies should be conducted to examine the direct effect of Mankai on health outcomes. Following a high-polyphenol diet, higher detection of plasma polyphenols and greater elevation in urine polyphenols and phenolic products were observed, suggesting an unbiased marker for polyphenol intake.

## Figures and Tables

**Table 1:** Examples of polyphenols detected in Mankai plant by class and subclass.

Polyphenol Class ^1^	Subclass ^1^	Examples of Polyphenols Detected in the Mankai Plant (Mankai Polyphenol Metabolomic Experiment ^#^)
Flavonoids	Flavonols	Quercetin (1,3,4,5), Rutin (1,4,5), Isorhamnetin (4,5), Kaempferol (3,4), Myricetin (4)
	Flavones	Apigenin (3,4,5), Luteolin (3,4,5), Orientin (1,4), Syringetin (4,5), Laricitrin (4)
	Flavanols	Epicatechin (4,5)
	Flavanones	Naringenin (4,5), Taxifolin (4,5), Astilbin (1)
Phenolic acids	Hydroxycinnamic acids	Caffeic acid (3,4,5), Ferulic acid (4,5), *p*-Coumaric acid (4,5), Sinapic acid (4)
	Hydroxybenzoic acids	Benzoic acid (4,5), Ellagic acid (4,5), Ginkgoic acid (1,2), Gallic acid (4,5), Vanillic acid (4,5), 4-Methylsalicylic acid (2)
Other polyphenols	Other polyphenols	Arbutin (4,5), Esculin (4,5)
	Hydroxycoumarins	Daphnetin (2,3,4), Coumarin (3)
	Phenolic terpenes	Carnosol (4)
Stilbenes	Stilbenes	*cis*-Resveratrol (4)
Unclassified		Olivetol (1,2)

^1^ Known class according to phenols explorer (USDA) or PubChem (NIH). ^#^ The number of Mankai polyphenol metabolomics experiment (1 = Mankai polyphenol metabolomic experiment 1, 2 = Mankai polyphenol metabolomic experiment 2, etc.).

**Table 2:** Baseline plasma polyphenols detection across DIRECT PLUS intervention groups.

Detected Polyphenols in Plasma, % ^1^	Entire *n* = 294	HDG *n* = 98	MED *n* = 98	Green-MED *n* = 98	*p* between Groups ^2^
Hippuric acid	93.0	94.6	89.4	94.9	0.25
m-hydroxyhippuric acid	82.8	80.7	84.0	83.5	0.80
Vanillin	61.3	61.3	60.6	61.8	0.99
2,6 diOH benzoic acid	45.4	47.3	43.6	45.4	0.88
3,4 hydroxyphenyl propionic acid	33.0	27.2	29.4	42.1	0.06
diOH isoferulic acid	28.5	23.7	27.7	34.0	0.28
Pyrogallol	20.8	18.3	22.3	21.7	0.77
Vanillic acid	15.5	15.1	13.8	17.5	0.77
2,5 diOH benzoic acid	9.2	6.5	8.5	10.3	0.63
Naringenin	0.35	0	0	1.03	0.38

^1^ Detected % is calculated of the total number of participants in each group, as reported in this table. ^2^ According to the chi-square test. diOH, dihydroxy.

## Data Availability

Data is contained within the article or Supplementary Material.

## Supplementary Materials

The following are available online at https://www.mdpi.com/article/10.3390/nu13061866/s1. Methods S1: Further information on Plant metabolites analyses. Methods S2: plasma polyphenol analysis. Methods S3: Urine polyphenols analysis. Table S1: Identification of major polyphenols from fresh Wolffia globosa ‘Mankai’ (Experiment 3). Table S2: A list of polyphenols detected in urine. Figure S1: Supplemental Figure S1: Annotation of major polyphenols in fresh Wolffia globosa ‘Mankai’ extract (Experiment3).
