# Supplementary material for: The Metabolomic-Gut-Clinical Axis of Mankai Plant-Derived Dietary Polyphenols
Source: Nutrients. 2021 May 30;13(6):1866. doi: 10.3390/nu13061866 (PMC8229908; doi:10.3390/nu13061866)
Supplement: Supplementary file 1 [file nutrients-13-01866-s001.zip › nutrients-1235691-supplementary.pdf]

The metabolomic-gut-clinical axis of Mankai plant derived dietary polyphenols

Supplemental Methods 1: Further information on Plant metabolites analyses

LC-QtoF-MS and LC-Orbitrap_MS HPLC (UFZ, Germany)- Mankai polyphenol metabolomics experiments 1+2

Extraction protocol: Extraction of the samples was performed following the protocol from De Vos et al.[1]. First, the dry samples were mixed with 300 µL of Methanol:Water (75:25) containing 0.1% Formic Acid. The samples were homogenized using a TissueLyser II (from QIAGEN GmbH, Germany) for 10 min at 30 Hrz. The extracts were vortexed and sonicated for 15 min at room temperature and then centrifuged for 15 min at 20,000g. The supernatant was taken and filtered through 0.2 mm PTFE filters and transferred in vials compatible with the LC autosampler.

LC-QtoF-MS: All the chemical screening was carried out with HPLC-QToF instrument from Agilent Technologies (6540 UHD Accurate-Mass Q-TOF LC/MS instrument). Every sample was injected twice in order to be ionized in positive and negative modes. 10 µL of sample was injected and loaded on a C18 column (an ACQUITY UPLC HSS T3, 2.1x100 mm column). The compounds were separated with a gradient of mobile phase A (0.1% formic acid in water) and mobile phase B (2% isopropanol, 0.1% formic acid in acetonitrile). The gradient was as follows: 0-5min 1% B, 5.1-20min 1%-100% B, 20.1-22min 100% B, 22.1-25min 1% B. The QToF was set up in centroid mode and in screening mode allowing the detection of ions with a mass to charge ratio between 60 and 1000 and every ions with an intensity above 200 counts was sent to the second MS in order to record their fragments.

LC-Orbitrap_MS HPLC-column and gradient were performed as stated above but on an Acquity Ultra Performance LC (Waters). The HPLC was coupled to an Orbitrap Velos mass spectrometer (Thermo Fisher Scientific) with a heated electrospray source. ESI heater temperature was set to 300°C. Full scan was acquired for m/z between 60 and 1000. The ten most intense signals were fragmented using CID with a collision energy of 35. Samples were measured in negative and positive ionization mode separately.

Processing of the LC-MS data: Following the principles described by Alonso et al. [2] the spectral processing was carried out using XCMS scripts [3] adapted for their use via Galaxy [4]. The workflow consisted in a pick picking step (using the xcmsSet script) following by a grouping step and retention time alignment (using xcmsGroup and xcmsRetcor scripts). The workflow finished with a fillPeak script and CAMERA annotate. This allowed the production of the feature matrix that was used for the statistical analysis. Before the statistics, the peaks were filtered by a blank subtraction and a normalization steps.

LC/MS (Weizmann, Israel) – Mankai polyphenol metabolomics experiment 3

Extraction protocol: Plants- 33 plant samples, 100-200mg each with 3 replicates, were filtrated, cold water washed and -80°C frozen before grinding with the TissueLyser Retsch mill (20Hz for 2min, x2). Extraction was done using 80% MeOH and 0.1% formic acid (w/v 1:3) by vortex followed by sonication for 20 min at 60Hz. The supernatant, separated by centrifugation at 17000g for 10 min at -4°C and filtration through 0.22μm PTFE, was injected for analysis on UPLC-QTOF-MS (40 min).

Metabolite annotation was performed either by matching to the WEIZMASS spectral library [5] or manually by calculation of elemental composition for molecular ions, matching of fragments from the high energy function and comparison between ionization modes.

Data analysis workflow was as follows: i. quality control (standard-mix samples, [x]); ii. XC-MS peak picking & quality control (EIC, Box, optimization); iii. Metabostat statistical analysis clustering; iv. Compounds identification (Weizmass, lab database, putative assignment).

Synapt G1 QTOF-MS and LC-MS/MS (Edmund Mach Foundation, Italy) – Mankai polyphenol metabolomics experiments 4+5

In "experiment 4", 0.1 g of Mankai powder was extract with 1 mL MeOH/H_2O/Formic acid (75/24.9/0.1) for 1 hour at room temperature with an orbital shaker, then centrifuged for 5 min and finally filtered and directly analyzed. In total 3 samples of Mankai, as powder, were extracted and analyzed. A Waters Acquity UPLC coupled via an electrospray ionization (ESI) interface to a Synapt G1 QTOF-MS (Waters, Manchester, UK) operating in W-mode and

controlled by MassLynx 4.1 was used. The UPLC conditions were developed in previously published methods (https://doi.org/10.1007/s11306-014-0638-x). All samples were analysed on a reversed phase (RP) ACQUITY UPLC 1.8 μm 2.1 x 150 mm HSS T3 column (Waters) at 40 °C and at a mobile phase flow rate of 0.28 mL/min. Water was used as weak eluting solvent (A) and methanol as strong eluting solvent (B); formic acid 0.1% v/v was used as additive in both eluents. The multistep linear gradient used was as follows: 0-1 min, 100% A isocratic; 1-3 min, 100-90 % A; 3-18 min, 90-60 % A; 18-21 min, 60-0 % A; 21-25.5 min, 0 % A isocratic; 25.5-25.6 min, 0-100 % A; 25.6-28 min 100% isocratic. Injection volume was 10 μL and the samples were kept at 4 ºC throughout the analysis. Mass spectrometric data were collected by separate runs in positive and negative ESI mode over a mass range of 50 to 2000 amu with scan duration of 0.3 s in centroid mode. The transfer collision energy and trap collision energy were set at 6 V and 4 V. The source parameters were set as follows: capillary 3 kV for positive scan and 2.5 kV for negative scan, sampling cone 25 V, extraction cone 3V, source temperature 150 ºC, desolvation temperature 500 ºC, desolvation gas flow 1000 L/h and nebulizer gas 50 L/h. External calibration of the instrument was performed at the beginning of each batch of analysis by direct infusion of a sodium formate solution (10% formic acid/0.1 M NaOH/Acetonitrile at a ratio of 1/1/8), controlling the mass accuracy from 40 to 2000 m/z (less than 5 ppm) and mass resolution (over 14000 FWHM). LockMass calibration was applied using a solution of leucine enkephaline (0.5 mg/L, m/z 556.2771 for positive and 554.2620 for negative ion mode) at 0.1 mL/min. In line between the LC and the MS instrument was used a DAD detector (Waters Acquity PDA) to register the UV spectra (240-400 nm) of the eluted metabolites. Metabolite annotation was achieved by using an internally build database (https://doi.org/10.1002/rcm.6705).

“Experiment 5” used 10 samples of different Mankai batches. 2 treatments: 5 samples →Sunlight, 5 samples → Led light.

Phenolic compounds were determined according to [6] with some modifications. Briefly, 1.6 mL of chloroform and 2.4 mL of methanol: water (2:1) were added to 100 mg of previousely ground dried Mankai leaf. A 20 μL aliquot of gentisic acid (50 mg/L) and rosmarinic acid (50 mg/L) were added as internal standards. The extraction mixture was shaken for 15 min in an orbital

shaker, then centrifuged for 5 min at 15,000 g at 4°C. The upper aqueous-methanolic phase was collected. The extraction was repeated by adding 2.4 mL of methanol and water (2:1 v/v) and 0.8 mL of chloroform. The samples were centrifuged for 5 min at 15,000 g at 4°C. The aqueous-methanolic phase was collected and combined with the previous one. The merged fractions were then evaporated to dryness under a gentle stream of N_2. Samples were, finally, re-suspended in 500 μL of methanol and water (1:1 v/v), and transferred carefully into an HPLC vial. Data processing was performed using Waters MassLynx 4.1 (Waters, Milford, CT, USA) and TargetLynx software (Waters, Milford, CT, USA). Details of the liquid chromatography and mass spectrometry are described in Vrhovsek et al. [6] and Gasperotti et al. [7].

Supplemental methods 2: plasma polyphenol analysis

A previously developed targeted metabolomic method was performed with an ultra-performance liquid chromatographic system coupled to a tandem mass spectrometry system with electrospray ionization (UHPLC-ESI-MS/MS). Before injection, samples were thawed at 4 °C. Sample preparation was performed using an Ostro™ Pass-through 96-well plate to remove phospholipids and proteins (Waters, Milford, MA, USA). An Ostro™ 96-well plate was fixed on top of a 96-well collection plate. 50 µl of plasma were pipetted into the wells, followed by the addition of 1% formic acid in acetonitrile (3:1 solvent/sample). The mixture was then quickly shaken for 5 minutes to promote protein precipitation. Vacuum (15 in. (~381 mm) Hg) was then applied to the Ostro plate through a vacuum manifold, filtering out the nonphospholipid plasma components. This step was repeated twice to ensure protein precipitation. Then, samples were dried under nitrogen and reconstituted in 100 µl of methanol: water (1:1, v/v), containing hippuric acid D5 (1 µg/ml) as an external standard. Samples were finally transferred to LC vials and injected (2 µL) into the UHPLC–MS/MS system. All solvents were kept at 4 °C prior to their use, and all procedures were carried out in a cold room, assuming that a 4 °C extraction temperature and the relatively short extraction time (10 min) may be favorable for avoiding biological sample degradation and reducing the risk of metabolite precipitation. Quality control (QC) samples were also prepared prior to analysis by pooling a small fraction of all the individual analyzed samples. Data processing was performed using Waters MassLynx

4.1 (Waters, Milford, CT, USA) and TargetLynx software (Waters, Milford, CT, USA). Details of the liquid chromatography and mass spectrometry were previously described [6] [7].

Supplemental Methods 3: Urine polyphenols analysis

Acetonitrile (ACN) and Water in LC-MS analytical grade were purchased from J.T. Baker (Part of Fisher Scientific). Formic acid was purchased from Honeywell (Charlotte, NC, USA) and β-glucuronidase (EC: 3.2.1.31) type HP-2 from *H.pomatia* was obtained from Sigma Aldrich (St.Louis, MO, USA).

Urine samples were thawed at RT for 5-10 min. For enzymatic deconjugation 15 µl β-glucuronidase were added to 50 µl urine and incubated for 2 h at 37 °C. Samples were then extracted twice by adding 600 µl ethyl acetate and shaking for 5 min at 1100 rpm. The organic phases were combined and centrifuged (15 min, 3000 rpm) to remove impurities. Subsequently, the supernatant was and dried in a SpeedVac™ vacuum concentrator (Eppendorf) and stored.

Prior to LC-MS/MS measurement the samples were resuspended in water with 1% ACN and 0.1% formic acid and 10 µl were injected into an HPLC-QToF instrument from Agilent Technologies (6540 UHD Accurate-Mass Q-TOF LC/MS instrument) Metabolites were loaded on a C18-precolumn (Acquity BEH C18 1.8 µm, 2.1 x 50 mm) separated on a C18 column (Acquity UPLC HSS T3 1.8 µm, 2.1 x 100 mm) at a flow rate of 0.3 mL/min with the following gradient of running solvent A (0.1% formic acid in water) and running solvent B (0.1% formic acid in acetonitrile): 0-5 min 1% B, 5.1-20 min 1%-100% B, 20.1-25 min 1% B. All samples were acquired in positive and negative ionization mode. The QToF was set up in centroid mode and in screening mode allowing the detection of ions with a mass to charge ratio between 60 and 1000. After each full scan, the 5 most intense ions (threshold 200 counts) were fragmented.

Raw files (.d) were imported into the Progenesis QI® software (v.2.1, Waters Corporation). Samples in different ionization modes were processed separately. The workflow included isotope and adduct fusion and chromatogram alignment in t_R direction based on a reference chromatogram was done. Next, peak picking was applied using default sensitivity settings. A database search was performed using ChemSpider as an identification method with the urine human metabolome database [8] and Phenol explorer [9] as input selection. Precursor and

fragment mass tolerance were set to 15 ppm and 10 ppm, respectively. Only precursor peaks with a corresponding fragment spectra were kept. Normalized peak areas and possible identifications were exported.

The exported possible feature identifications were filtered using in-house written R scripts [10], Briefly, feature identifications were filtered for Progenesis score of at least 40. Then for each feature, only the top scoring identification and those which had a score less than 5 lower than the top score was kept. Finally, the resulting filtered data were further analyzed using a second in-house written R-script to extract possible phenolic compounds in the identification list. Only identifications where the phenolic compound was the top-scoring hit or shared top scoring hit was annotated as a phenolic compound.

Supplemental Table S1: Identification of major polyphenols from fresh Wolffia globosa 'Mankai' (Experiment 3).

#	putative identification	RT [min]	λ_{max} [nm]	$[M-H]^-$	Δppm	$[M+H]^+$	Δppm	formula	fragments [ESI-]
**1**	caffeic acid hexose	3.75	300, 330	341.0864	-2.6	343.1042	3.8	$C_{15}H_{18}O_9$	179.0355, 161.0247, 133.031
**2**	coumaric acid hexose (*)	5.11	315	325.0908	-4.6	327.1088	2.4	$C_{15}H_{18}O_8$	163.0421, 145.0305, 117.0358
**3**	coumaric acid hexose	5.72	317	325.0919	-1.2	nd	nd	$C_{15}H_{18}O_8$	163.0419, 145.0314, 117.0363
**4**	Luteolin di hexose	6	336	609.1434	-3.6	611.1620	1.3	$C_{27}H_{30}O_{16}$	489.1017, 447,0909, 429.0825, 357.0608, 327.0501, 285.0470, 175.0418
**5**	Apigenin di hexose	6.1	271, 325	593.1498	-1.3	595.1657	-1.0	$C_{27}H_{30}O_{15}$	503.1175, 473.1072, 431.1081, 341.0670, 311.0542, 282.0525
**6**	Luteolin di hexose	6.18	270, 350	609.1472	2.6	611.1627	2.5	$C_{27}H_{30}O_{16}$	519.0891, 489.1029, 453.0640, 429.0691, 399.0703, 369.0609
**7**	Propenoic acid hexose	6.3	334	385.1125	-2.6	nd	nd	$C_{17}H_{22}O_{10}$	431.1916 (FA adduct), 223.0629, 205.0506, 190.0277, 175.0025, 119.0165
**8**	Apigenin di hexose	6.6	270, 341	593.1503	-0.5	595.1684	3.5	$C_{27}H_{30}O_{15}$	503.1194, 473.1061, 383.0750, 353.0664, 325.0788, 269.0435
**9**	Luteolin di hexose	6.66	269, 350	609.1448	-0.8	611.1616	0.7	$C_{27}H_{30}O_{16}$	411.0699, 393.0595, 369.0618, 357.0595, 339.0529, 327.0477, 299.0544, 285.0405
**10**	Quercetin hexose	7.2	300, 327	593.1494	-2	595.1655	-1.3	$C_{27}H_{30}O_{15}$	nd

11	Flavonoid hexose pentose	7.49	270, 349	579.1337	-1.3	581.1514	1.4	$C_{26}H_{28}O_{15}$	519.1135, 489.1022, 459.0906, 429.0823, 399.0706, 369.0609
12	Apigenin hexose pentose	7.62	271, 350	579.1338	-1.2	581.1515	1.5	$C_{26}H_{28}O_{15}$	519.1199, 489.1031, 459.0910, 441.0818, 429.0800, 399.0683, 369.0616
13	unknown flavonoid	7.8	350	579.1353	0.5	581.1511	0.9	$C_{26}H_{28}O_{15}$	nd
14	Flavonoid hexose pentose	7.98	270, 340	579.1335	1.7	581.1517	1.9	$C_{26}H_{28}O_{15}$	519.1245, 489.1065, 459.0904, 429.0789, 399.0704, 369.0615
15	Apigenin di hexose	7.98	270, 340	593.1517	1.1	595.1664	0.2	$C_{27}H_{30}O_{15}$	341.0666, 311.0552, 269.0464
16	Apigenin hexose pentose	8.12	266, 342	563.1393	-1.4	565.1579	3.9	$C_{26}H_{28}O_{14}$	443.0926, 383.0726, 353.0681
17	Quercetin 3,4'-diglucoside (*)	8.14	266, 342	625.1397	-0.8	627.1572	1.8	$C_{27}H_{30}O_{17}$	463.0853, 301.0359, 255.0314
18	8-C-Galactosylluteolin (*)	8.42	269, 349	447.0928	0.2	449.1075	-2.0	$C_{21}H_{20}O_{11}$	895.1938 (dimer), 429.0801, 357.0585, 327.0484, 297.0399, 285.0408, 255.0302
19	Apigenin-6,8-C-hexosyl-8-C-pentose (*)	8.53	271, 336	563.1377	-4.3	565.1569	2.1	$C_{26}H_{28}O_{14}$	1127.2920 (dimer), 545.1286, 485.1107, 473.1091, 455.0891, 443.0966, 425.0865, 413.0879, 395.0774, 383.0738, 365.0653, 353.0635, 337.0663, 325.0675, 297.0699
20	Apigenin hexose pentose	8.62	271, 335	563.1422	3.7	565.1563	1.1	$C_{26}H_{28}O_{14}$	1127.2914 (dimer), 545.1312, 503.1198, 485.1080, 473.1090, 455.0936, 443.0959, 425.0885, 413.0861, 395.0782, 383.0743, 365.0667, 353.0650, 337.0746, 325.0699, 297.0729
21	8-hexosyl-luteolin	8.79	270, 349	447.0936	2	449.1077	-1.6	$C_{21}H_{20}O_{11}$	895.1940 (dimer), 393.0612, 369.0604, 357.0596, 327.0474, 297.0403, 285.0395, 255.0413
22	unknown flavonoid	9	nd	563.1409	1.4	565.1581	4.2	$C_{26}H_{28}O_{14}$	327.049
23	coumaric acid derivative (*)	9.21	313	279.0512	0.6	281.0671	3.6	$C_{13}H_{12}O_7$	559.1132 (dimer), 163.0415, 119.0517
24	Apigenin hexose pentose	9.42	269, 325	563.1426	4.4	565.1578	3.7	$C_{26}H_{28}O_{14}$	535.1923, 503.1635, 473.1129, 443.1058, 413.0757, 395.0804, 383.0761, 353.0660, 325.0703, 297.0686
25	coumaric acid derivative	9.66	309	279.0525	7.5	nd	nd	$C_{13}H_{11}O_7$	163.0415, 133.0156, 119.0517
26	Apigenin-6-C-Hexose (*)	9.98	268, 339	431.0933	-10.4	433.1126	-2.1	$C_{21}H_{20}O_{10}$	341.0655, 311.0532, 283.0605, 269.0432

27	Apigenin hexose	10.11	270, 339	431.0948	-7	433.1147	2.8	$C_{21}H_{20}O_{10}$	341.0661. 311.0540, 283.0603, 269.0470
28	Isoquercitrin (*)	10.47	353	463.0854	-5	465.1048	3.2	$C_{21}H_{20}O_{12}$	927.1909 (dimer)
29	Luteolin hexose (*)	10.78	270, 349	447.0901	-5.8	449.1084	-2.7	$C_{21}H_{20}O_{11}$	895.1948 (dimer), 337.0497, 285.0385, 255.0285

Characteristics 29 identified compounds (putative identifications) are shown with putative name, retention time, absorption, accurate mass in negative and positive ionization mode with mass error compared to theoretical mass of the displayed elemental composition/formula. Fragmentation spectra acquired in negative ionization mode were used for identification and major compound specific fragments are shown. Compounds marked with (*) were confirmed by comparison to Weizmass natural product library (Shahaf et al. 2016). Abbreviations: #: number, RT: retention time, λmax [nm]: absorption maximum in nanometer, M: molecular mass, H: proton, Δppm: mass error in parts per million, ESI-: Electron spray ionization in negative polarity.

Supplemental Table S2: A list of polyphenols detected in urine

n100-Dihydrosinapic_acid_(968)
n101-Hydroferulic acid_(966)
n105-3-Hydroxy-5-{(E)-2-[4-(sulfooxy)phenyl]vinyl}phenyl hydrogen sulfate_(1003)
n108-Hydroferulic acid_(966)
n111-m-hydroxy-Hydrocinnamic acid_or_4-hydroxyhydratropic acid_(961_978)
n116-Hydroxyphenylacetic_acid_(573_952_953)
n120-(E)-Isoferulic acid_or_Ferulic acid_(459_485)
n122-(2E)-3-[4-Methoxy-3-(sulfooxy)phenyl]acrylic acid_(937)
n128-5-(3,5-Dihydroxybenzyl)dihydro-2(3H)-furanone_(985)
n131-Hydroferulic acid_(966)
n134-5-(3,4-Dihydroxyphenyl)pentanoic_acid_(982)
n137-Urolithin C_(1040)

n141-4-[(Z)-2-(3,5-Dihydroxyphenyl)vinyl]phenyl_beta-D-glucopyranosiduronic_acid_(997)
n142-4-(2-Hydroxyethyl)phenyl hydrogen sulfate_(1050)
n144-Norathyriol_(1060)
n148-2-[4-Hydroxy-3-(3-hydroxybenzyl)-2-(hydroxymethyl)butyl]-1,4-benzenediol_(1024)
n162-5-(3,4,5-Trihydroxybenzyl)dihydro-2(3H)-furanone_(984)
n163-Tyrosol_(673)
n165-4-(2-Hydroxyethyl)phenyl hydrogen sulfate_(1050)
n169-(4R)-3-[(S)-Hydroxy(4-hydroxy-3-methoxyphenyl)methyl]-4-(4-hydroxy-3-methoxybenzyl)dihydro-2(3H)-furanone_(614)
n174-5-(3,4-Dihydroxyphenyl)-4-hydroxypentanoic acid_(981)
n176-Tyrosol_(673)
n177-4-(2-Hydroxyethyl)phenyl hydrogen sulfate_(1050)
n179-Salicylic_acid_(428)
n187-2-[4-Hydroxy-3-(3-hydroxybenzyl)-2-(hydroxymethyl)butyl]-1,4-benzenediol_(1024)
n190-Daidzein_(394)
n192-5-(3,4,5-Trihydroxybenzyl)dihydro-2(3H)-furanone_(984)
n193-Urolithin A_(1037)
n195-(E)-Isoferulic acid_or_Ferulic acid_(459_485)
n197-Dihydrodaidzein_(861)
n199-Tyrosol_(673)
n204-4-(2-Hydroxyethyl)phenyl hydrogen sulfate_(1050)
n207-Quercetin_(291)
n216-2-[4-Hydroxy-3-(3-hydroxybenzyl)-2-(hydroxymethyl)butyl]-1,4-benzenediol_(1024)
n229-4-(5,7-Dihydroxy-6-methoxy-4-oxo-4H-chromen-3-yl)phenyl_hydrogen_sulfate_(887)

n233-Phloretin_(108)
n235-Naringenin_(201)
n236-Homovanillyl_alcohol_(643)
n240-4-[(E)-2-(3,5-Dihydroxyphenyl)vinyl]phenyl_hydrogen_sulfate_or_Resveratrol-3-O-Sulfate_(998_1000_1007)
n248-5,7-Dihydroxy-3-(2-hydroxy-4-methoxyphenyl)-8-methoxy-4H-chromen-4-one_(891)
n254-Isorhamnetin_(318)
n259-(3S,4S)-3-(4-Hydroxyphenyl)-4,7-chromanediol_(907)
n264-4-[-2-(3,5-Dihydroxyphenyl)vinyl]phenyl_hydrogen_sulfate_(998_1007)
n270-dalbergin_(919)
n271-5-(3,4-Dihydroxyphenyl)pentanoic_acid_(982)
n279-MW5143500_(579)
n280-Hydroferulic acid_(966)
n281-Hydroferulic acid_(966)
n290-Isosakuranetin_(218)
n292-6-[(1R,3aR,4S,6aR)-4-(1,3-Benzodioxol-5-yl)tetrahydro-1H,3H-furo[3,4-c]furan-1-yl]-1,3-benzodioxol-5-ol_(627)
n299-7-Hydroxy-3-(4-methoxyphenyl)-2,3-dihydro-4H-chromen-4-one_(863)
n304-Eriodictyol_(202)
n305-(3S)-3-(4-Hydroxy-3-methoxyphenyl)-7-chromanol_(901)
n355-Gallic acid_(413)
n359-(3R,4R)-4_or_3-(2,5-Dihydroxybenzyl)-3_or_4-(3-hydroxybenzyl)dihydro-2(3H)-furanone_(1027_1030)
n373-Phenylacetic_acid_(956)

n409-Gallic acid_(413)
n420-(2R,3S)-2-(4-Hydroxy-3-methoxyphenyl)-3,5,7-chromanetriol_or_4'_or_3'-METHYLEPICATECHIN_(766_770_775)
n421-2,4-Dihydroxybenzoic acid_or_gentisic acid_(412_416_431_416_436)
n427-(?)-Epicatechin_or_D-(+)-Catechin_(125_126)
n44-Phenylacetic_acid_(956)
n445-3-Methoxy-4-hydroxyhippuric acid_(1064)
n447-Hydroxytyrosol_(674)
n453-3-Hydroxy-5-{(E)-2-[4-(sulfooxy)phenyl]vinyl}phenyl hydrogen sulfate_(1003)
n466-Homovanillic acid_(574)
n468-cis-Resveratrol_3-O-glucuronide_(995)
n471-3-Methoxy-4-hydroxyhippuric acid_(1064)
n477-AM7525000_(1059)
n483-Dihydroxybenzoic acid_or_gentisic acid_()
n484-Gallic acid_()
n492-Ethyl gallate_(439)
n500-Hippuric_acid_(929)
n504-3-Methoxy-4-hydroxyhippuric acid_(1064)
n509-(2E)-3-[4-Methoxy-3-(sulfooxy)phenyl]acrylic acid_(937)
n52-Gallic acid_(413)
n60-3,4-Dihydroxy-5-methoxybenzoic_acid_or_3,5-Dihydroxy-4-methoxybenzoic_acid_(922_923)
n65-Hydroxytyrosol_(674)
n69-3-Methoxy-4-hydroxyhippuric acid_(1064)
n73-2,4-Dihydroxybenzoic acid_or_gentisic acid_(431_416)

n76-2,4-Dihydroxybenzoic acid_or_gentisic acid_(431_416)
n78-O-feruloyl-D-quinic_acid_or_3-Feruloylquinic_aci_(478_479_480)
n81-Caffeic_acid_3-sulfate_or_(2E)-3-[3-Hydroxy-4-(sulfooxy)phenyl]acrylic_acid_(479_941)
n85-Homovanillic_acid_or_Coumaric_acid_or_MW5143500_(454_463_574_579)
n93-5-(3,5-Dihydroxybenzyl)dihydro-2(3H)-furanone_(985)
n95-Vanillic acid_(414)
n96-(2E)-3-[4-Methoxy-3-(sulfooxy)phenyl]acrylic acid_(937)
p125-3-(4-Hydroxyphenyl)-4-oxo-3,4-dihydro-2H-chromen-7-yl beta-D-glucopyranosiduronic acid_(862)
p127-p_or_m-Hydroxyhippuric_acid_(926_927)
p135-Tyrosol_(673)
p165-Isoferulic_or_Ferulic_acid_(459_485)
p166-Hydroferulic acid_(966)
p177-Urolithin C_(1040)
p198-Hydroferulic acid_(966)
p199-4-Ethylphenol_(707)
p200-Schisantherin A_(1017)
p203-Homovanillyl alcohol_(643)
p218-Oleuropein_(677)
p219-5-(3,4-Dihydroxyphenyl)-4-hydroxypentanoic acid_(981)
p232-Naringenin_or_Daidzein_(201_394)
p234-3-Phenylpropanoic acid_(975)
p235-Urolithin A_(1037)
p237-Tyrosol_(673)

p240-Dihydrodaidzein_(861)
p247-(3R,4R)-4-(2,5-Dihydroxybenzyl)-3-(3-hydroxybenzyl)dihydro-2(3H)-furanone_(1030_1027)
p253-3-(4-Hydroxyphenyl)-6-methoxy-7-chromanol_(836_902)
p254-Melannin_(915)
p256-Kaempferol_(290)
p260-(3R,4R)-4-(2,5-Dihydroxybenzyl)-3-(3-hydroxybenzyl)dihydro-2(3H)-furanone_(1030)
p272-(2Z)-3-[2-(Carboxymethyl)-3,4-dihydroxyphenyl]acrylic acid_(1045)
p275-Homovanillyl alcohol_(643)
p277-p-Hydroxymandelic acid_(957)
p295-Eriodictyol_(202)
p296-Naringenin_(201)
p301-5-(3',5'-Dihydroxyphenyl)-gamma-valerolactone 3-O-glucuronide_(988)
p311-3-Phenylpropanoic acid_(975)
p315-Hydroferulic acid_(966)
p318-3-Phenylpropanoic acid_(975)
p347-dalbergin_(919)
p356-4-[(3S)-7-Hydroxy-3,4-dihydro-2H-chromen-3-yl]phenyl beta-D-glucopyranosiduronic acid_(894_893)
p358-6-[(1R,3aR,4S,6aR)-4-(1,3-Benzodioxol-5-yl)tetrahydro-1H,3H-furo[3,4-c]furan-1-yl]-1,3-benzodioxol-5-ol_(627)
p366-Tectorigenin_or_psi-tectorigenin_(889_892)
p368-(2Z)-3-[2-(Carboxymethyl)-3,4-dihydroxyphenyl]acrylic acid_(1045)
p370-(4R)-3-[(S)-Hydroxy(4-hydroxy-3-methoxyphenyl)methyl]-4-(4-hydroxy-3-methoxybenzyl)dihydro-2(3H)-furanone_(614)

p389-Hesperetin_or_Homoeriodictyol_(203_795)
p390-irisolidone_(888)
p406-3-(4-Hydroxyphenyl)-6-methoxy-7-chromanol_(836_901_902)
p410-Hydroferulic acid_(966)
p454-Tyrosol_(673)
p47-Benzoic acid_(427)
p48-Hippuric acid_(929)
p545-4-Ethylphenol_(707)
p599-3-Methoxy-4-hydroxyhippuric acid_(1064)
p611-p_or_m-Hydroxyhippuric_acid_(926_927)
p625-3-Methoxy-4-hydroxyhippuric acid_(1064)
p641-3-Phenylpropanoic acid_(975)
p649-3,4-dihydroxyphenylacetic acid_(572)
p663-3-Methoxy-4-hydroxyhippuric acid_(1064)
p672-Dihydroxybenzoic acid_or_gentisic_acid_(431_436_430_416_412)
p690-p_or_m-Hydroxyhippuric_acid_(926_927)
p78-Isoferulic_or_Ferulic_acid_(459_485)

Supplemental Figure S1: Annotation of major polyphenols in fresh Wolffia globosa 'Mankai' extract (Experiment3).

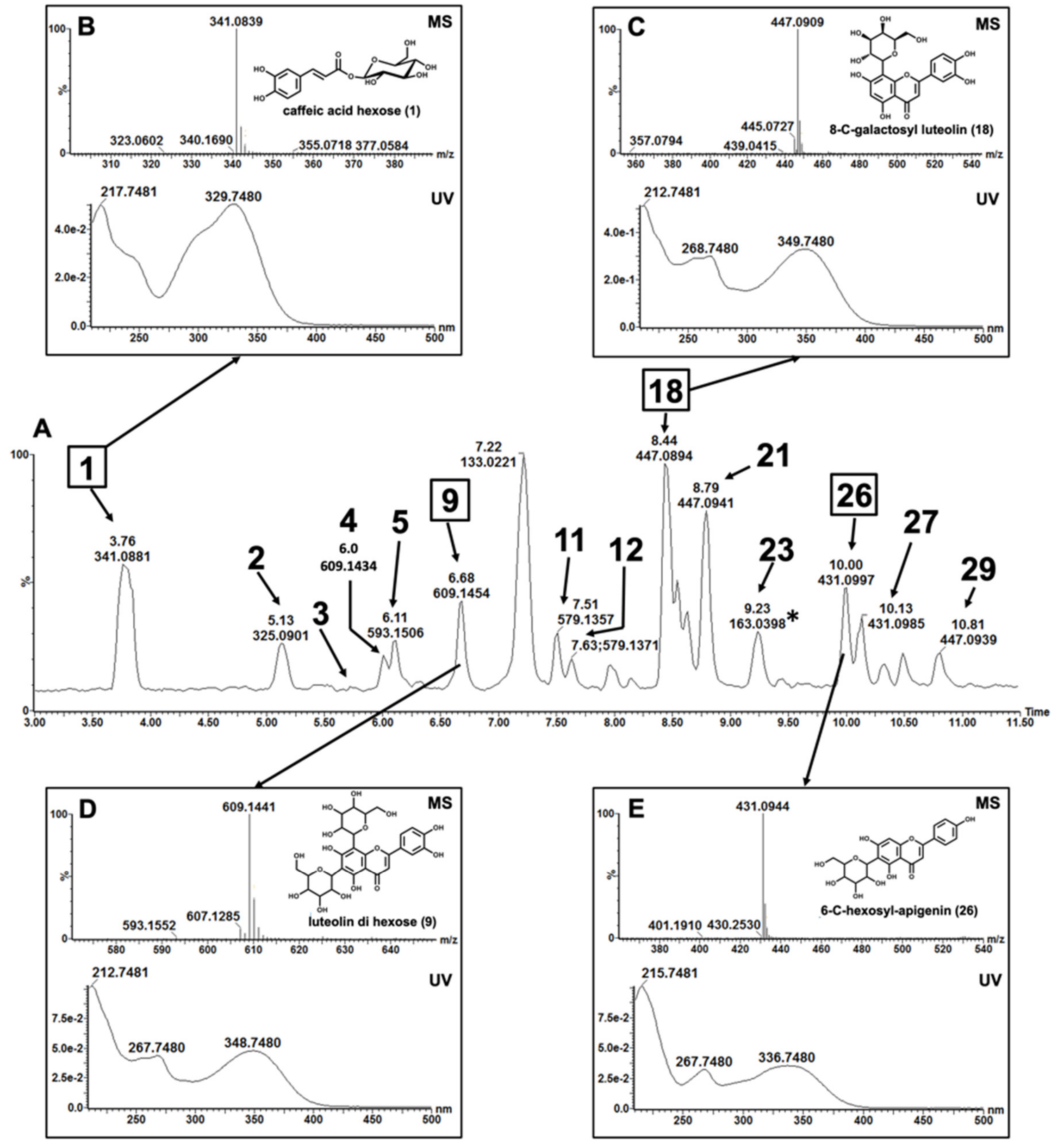

A: Total ion chromatogram acquired in negative ionization mode (ESI-) and numbers of compounds according to table 2. For each peak accurate mass of [M-H]- and retention time in minutes are displayed. * m/z 163.0398 is not the molecular ion, but the coumaric acid fragment which is originated from m/z 279.0525. B: mass spectrum, UV spectrum and putative structure

of metabolite 1 (caffeic acid hexose). C: mass spectrum, UV spectrum and putative structure of metabolite 18 (8-C-galactosyl luteolin). D: mass spectrum, UV spectrum and putative structure of metabolite 9 (luteolin di hexose). mass spectrum, UV spectrum and putative structure of metabolite 26 (6-C-hexosyl apigenin).

References

1. De Vos, R.C.H.; Moco, S.; Lommen, A.; Keurentjes, J.J.B.; Bino, R.J.; Hall, R.D. Untargeted large-scale plant metabolomics using liquid chromatography coupled to mass spectrometry. *Nat. Protoc.* **2007**, *2*, 778.
2. Alonso, A.; Marsal, S.; Julià, A. Analytical methods in untargeted metabolomics: state of the art in 2015. *Front. Bioeng. Biotechnol.* **2015**, *3*, 23.
3. Smith, C.A.; Want, E.J.; O'Maille, G.; Abagyan, R.; Siuzdak, G. XCMS: processing mass spectrometry data for metabolite profiling using nonlinear peak alignment, matching, and identification. *Anal. Chem.* **2006**, *78*, 779–787.
4. Giacomoni, F.; Le Corguillé, G.; Monsoor, M.; Landi, M.; Pericard, P.; Pétéra, M.; Duperier, C.; Tremblay-Franco, M.; Martin, J.-F.; Jacob, D. Workflow4Metabolomics: a collaborative research infrastructure for computational metabolomics. *Bioinformatics* **2015**, *31*, 1493–1495.
5. Shahaf, N.; Rogachev, I.; Heinig, U.; Meir, S.; Malitsky, S.; Battat, M.; Wyner, H.; Zheng, S.; Wehrens, R.; Aharoni, A. The WEIZMASS spectral library for high-confidence metabolite identification. *Nat. Commun.* **2016**, *7*, 1–13.
6. Vrhovsek, U.; Masuero, D.; Gasperotti, M.; Franceschi, P.; Caputi, L.; Viola, R.; Mattivi, F. A versatile targeted metabolomics method for the rapid quantification of multiple classes of phenolics in fruits and beverages. *J. Agric. Food Chem.* **2012**, *60*, 8831–8840.
7. Gasperotti, M.; Masuero, D.; Guella, G.; Mattivi, F.; Vrhovsek, U. Development of a targeted method for twenty-three metabolites related to polyphenol gut microbial metabolism in biological samples, using SPE and UHPLC–ESI-MS/MS. *Talanta* **2014**, *128*,

221–230.

8. Wishart, D.S.; Feunang, Y.D.; Marcu, A.; Guo, A.C.; Liang, K.; Vázquez-Fresno, R.; Sajed, T.; Johnson, D.; Li, C.; Karu, N. HMDB 4.0: the human metabolome database for 2018. *Nucleic Acids Res.* **2018**, *46*, D608–D617.

9. Rothwell, J.A.; Perez-Jimenez, J.; Neveu, V.; Medina-Remon, A.; M'Hiri, N.; García-Lobato, P.; Manach, C.; Knox, C.; Eisner, R.; Wishart, D.S. Phenol-Explorer 3.0: a major update of the Phenol-Explorer database to incorporate data on the effects of food processing on polyphenol content. *Database* **2013**, *2013*.

10. Ihaka, R.; Gentleman, R. R: a language for data analysis and graphics. *J. Comput. Graph. Stat.* **1996**, *5*, 299–314.
